# Unraveling the earliest phases of vascular cognitive impairment and dementia by establishing a large USA CADASIL Consortium

**DOI:** 10.1002/alz.093152

**Published:** 2025-01-09

**Authors:** Jane S Paulsen, Suman Jayadev, Michael D Geschwind, Jose Biller, Jamie L Elliott, Jose Gutierrez, Sudha Seshadri, Jennifer J Majersik, Karen D Orjuela, David S Liebeskind, Helmi L Lutsep, Ihab Hajjar, Henry J Bockholt, Stephen Salloway

**Affiliations:** ^1^ University of Wisconsin‐Madison, Madison, WI USA; ^2^ University of Washington, Seattle, WA USA; ^3^ University of California, San Francisco (UCSF), San Francisco, CA USA; ^4^ Loyola University Chicago, Chicago, IL USA; ^5^ Columbia University, New York, NY USA; ^6^ University of Texas Health Science Center at San Antonio, San Antonio, TX USA; ^7^ University of Utah, Salt Lake City, UT USA; ^8^ University of Colorado Anschutz Medical Campus, Aurora, CO USA; ^9^ University of California‐Los Angeles, Los Angeles, CA USA; ^10^ Oregon Health & Science University, Portland, OR USA; ^11^ UT Southwestern Medical Center, Dallas, TX USA; ^12^ Tri‐Institutional Center for Translational Research in Neuroimaging and Data Science (TReNDS), Atlanta, GA USA; ^13^ Butler Hospital, Providence, RI USA

## Abstract

**Background:**

CADASIL, linked to NOTCH3 variants, is a primary monogenic cause of vascular dementia, leading to vascular cognitive impairment and dementia (VCID) observable in early stages. The NIH‐funded USA CADASIL Consortium aims to explore CADASIL's onset and progression in the USA, crucial due to varying phenotype‐genotype associations globally. The consortium will identify biological and clinical markers across the disease spectrum, contributing to clinical trial preparations. It will focus on clinical outcome assessments, biofluids and neuroimaging for biomarker identification, and disease modeling for subtypes, informing future clinical trial designs. The study will also examine genetic, health, and lifestyle factors impacting CADASIL outcomes.

**Method:**

The methodology involves establishing a longitudinal cohort of 400 NOTCH3 carriers and 100 non‐carrier family members across 12 USA sites (www.cadasil‐consortium.org). Participants undergo annual clinical, cognitive assessments, advanced neuroimaging, and blood‐based phenotyping, targeting optimal biomarkers to determine minimal sample sizes for clinical trials.

**Results:**

Despite COVID‐19 challenges, over 200 participants are enrolled, many already in longitudinal assessments. The Symptomatic (SYM) group, individuals with mild to moderate disability (Rankin Scale scores 1‐3), displays different cognitive and functional patterns from the Non‐Symptomatic (NSYM) group. The SYM group, mostly females (63) and Caucasians (101), has lower MOCA scores (average 25.2 vs. NSYM's 26.8) and SDMT averages (43.7 vs. NSYM's 51.4), indicating reduced cognitive function and processing speed. Functionally, SYM's average WHODAS score is 9.1, higher than NSYM's 3.1. The SYM group, averaging 53 years, is older than NSYM's 47.7 years, highlighting age's role in symptom severity.

**Conclusion:**

The upcoming AAIC presentation will provide a comprehensive overview of the US CADASIL cohort, contrasting it with international data. Efforts are ongoing to establish a worldwide CADASIL Study Group to advance knowledge and treatment for vascular cognitive impairment and dementias.

